# Supplementary material for: Association between oral microbiome and seven types of cancers in East Asian population: a two-sample Mendelian randomization analysis
Source: Front Mol Biosci. 2023 Nov 21;10:1327893. doi: 10.3389/fmolb.2023.1327893 (PMC10702768; doi:10.3389/fmolb.2023.1327893)
Supplement: Supplementary file 1 [file DataSheet1.ZIP › Supplementary Materials/MR plots for tongue/tongue═╝/Prostate cancer/pheno.315_to_prostate cancer_forest.pdf]

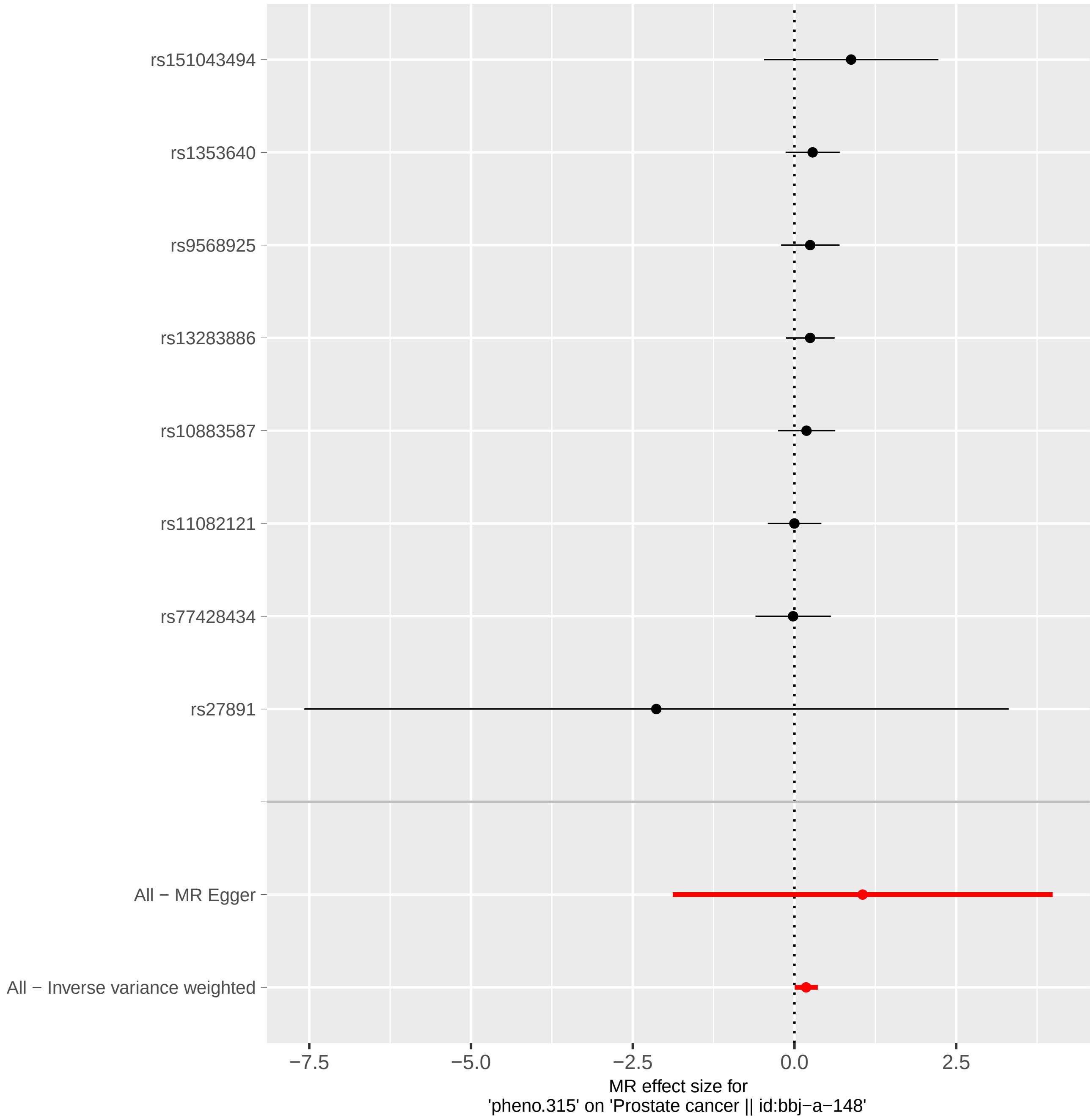
rs151043494
rs1353640
rs9568925
rs13283886
rs10883587
rs11082121
rs77428434
rs27891
All − MR Egger
All − Inverse variance weighted
−7.5
−5.0
−2.5
0.0
2.5
MR effect size for
'pheno.315' on 'Prostate cancer || id:bbj−a−148'
